# An evaluation of the Index4 tool for chemotherapy toxicity prediction in cancer patients older than 70 years old

**DOI:** 10.1038/s41598-023-28309-5

**Published:** 2023-01-19

**Authors:** Alexis Lewis, Melissa Reed, Natalie Walde, Ioannis A. Voutsadakis

**Affiliations:** 1 Clinical Trials Unit, Sault Area Hospital, Sault Ste Marie, ON Canada; 2 Algoma District Cancer Program, Sault Area Hospital, 750 Great Northern Road, Sault Ste Marie, ON P6B 0A8 Canada; 3 Section of Internal Medicine, Division of Clinical Sciences, Northern Ontario School of Medicine, Sudbury, ON Canada

**Keywords:** Biomarkers, Medical research

## Abstract

Chemotherapy, although beneficial for improving outcomes in both localized and metastatic cancers, may be associated with significant adverse effects, especially for patients with decreased functional reserves. Prediction of patients who will not tolerate well chemotherapy treatment may help in modifying treatment plans and in reallocating resources to vulnerable patients. One hundred seventeen consecutive cancer patients over the age of 70 scheduled for chemotherapy treatment in a single cancer center were included in the study. Prediction of adverse chemotherapy outcomes were calculated using a prediction tool proposed and validated from the Cancer and Aging Research Group (CARG) and a prediction tool proposed by us, called Index4. The 2 tools were compared for their ability to predict grade 3 and 4 toxicities, Emergency Department (ED) and hospital admissions and chemotherapy discontinuation. The accuracy of both predictive tools was suboptimal. A high CARG score had a sensitivity of 46.3% and a specificity of 82% and an Index4 of 1 or above had a sensitivity of 53.7% and a specificity of 60% in predicting grade 3–4 adverse effects. The performance of the 2 tools in predicting ED and hospital admissions and chemotherapy discontinuation was comparable. An Index4 score of 0 was superior in predicting absence of grade 3–4 toxicities than a low CARG score (p = 0.002, McNemar’s test). The CARG tool for chemotherapy adverse effect prediction in geriatric cancer patients and the Index4 were able to predict adverse outcomes with moderate accuracy. Given its ease of calculation Index4 may be an alternative to CARG tool, suitable for a busy oncology practice.

## Introduction

Despite increasing use of targeted therapies and immunotherapies in cancer treatment, chemotherapy remains an important part of the systemic therapy of cancer both in the adjuvant and in the palliative setting. Most common cancers, including breast cancer, prostate cancer and lung cancers can often be treated effectively with hormonal therapies and other targeted therapies or with immunotherapies. In other cases the most effective or the only option is chemotherapy with its associated toxicities.

Adverse effects of chemotherapy tend to be more severe in patients with comorbidities or with decreased general status^1^. Older patients have a higher burden of comorbidities and often a compromised performance status, making administration of chemotherapy treatments more challenging. However, many older cancer patients are less affected by comorbidities and are functionally intact. Thus, they may be able to undergo more intense therapies with acceptable adverse effects and derive oncologic benefits similar to younger patients. With increasing age, the majority of cancer patients, though, develop some degree of functional and cognitive reserves decline that can be unmasked and accelerated by their cancer and its treatments, especially if it has been unrecognized^2^.

Evaluation of geriatric cancer patients with a geriatric assessment has been advocated as a means of identifying vulnerabilities of elderly oncologic patients and better adjust therapies to the individual patient status^3,4^. For prediction of chemotherapy toxicity in elderly patients a guideline from the American Society of Clinical Oncology (ASCO) recommends one of two available tools, the Cancer and Aging Research Group (CARG) tool or the Chemotherapy Risk Assessment Scale for High Age patients (CRASH) tool^5,6^. The score of the CARG tool is calculated by taking into consideration 11 factors of the patient, the cancer, laboratory parameters and functional data^5^. Despite its proven usefulness the CARG tool remains fairly complicated and unpractical for implementation in the every-day oncology practice. Tools that are more practical and reliably predict severe grade toxicity as well as other adverse outcomes of chemotherapy in older cancer patients are needed. These would be easier to implement and would possibly have a higher utility in the framework of oncology practices with limited resources.

We had previously proposed an easy to calculate predictive tool, called Index4, based on four parameters available or routinely obtainable at baseline for most cancer patients^7^. Index4 uses patient’s performance status, stage of cancer and two laboratory parameters (albumin and creatinine clearance) for calculation of an overall score. Cancer patients over age 70 with an Index4 score of 1 or above were shown to have grade 3 or 4 toxicities at a rate of 81.4% while grade 3 or 4 toxicities developed in 62% of patients with Index4 score equal to 0^7^. In addition, hospitalization rate was 49% in patients with Index4 score of 1 or above and 23% in patients with Index4 score equal to 0. In the current paper, we examine the performance of Index4 tool and compare it to the CARG tool for prediction of chemotherapy toxicity, hospitalizations, ED visits and chemotherapy discontinuation in a prospective cohort of cancer patients above age 70.

## Methods

Consecutive patients starting adjuvant chemotherapy for a localized cancer or palliative first line chemotherapy for a metastatic cancer in a single cancer center were enrolled in the study over a 2 year period. Other inclusion criteria were age above 70 and a diagnosis of cancer confirmed histologically. Exclusion criteria were patients’ age younger than 70 years old, and previous chemotherapy in the adjuvant or metastatic setting. Planned concomitant radiotherapy with the chemotherapy was allowed. Immunotherapies or targeted therapies in combination with chemotherapy were allowed, but patients receiving immunotherapy or targeted therapies without chemotherapy were excluded. Demographic data, and tumor clinicopathologic data were extracted from patient records. Clinical and laboratory information enabling calculation of baseline index4 and the Cancer and Aging Research Group (CARG) risk were extracted from charts and recorded. Index4 is calculated by adding 1 point each for ECOG performance status (PS) above 1, creatinine clearance below 40 mL/min, albumin below 35 g/L and stage 4 cancer (Supplemental Table 1)^7^. For the calculation of CARG risk category, several parameters are taken into consideration and are attributed 1 to 3 points^5^. A highest weight with attribution of 3 points each are assigned to hemoglobin below 11 g/dL for men or 10 g/dL for women, creatinine clearance below 34 mL/min, and 1 or more falls in the last 6 months (Supplemental Table 1). Two points each are given for age older than 72 years old, gastrointestinal or genitourinary cancer type, standard dose of chemotherapy dose (as opposed to reduced dose), polychemotherapy (as opposed to chemotherapy monotherapy), hearing self-evaluated as fair or worse, and severe or somewhat limited ability to walk 1 block. A lower weight with 1 point each are allocated if the patient needs help to take medications and for decreased social activity. A CARG low risk is defined as a total score of 0 to 5, intermediate risk is defined as a score of 6 to 9 and CARG high risk is defined as a score of 10 or above. Chemotherapy treatment regimen and tolerability outcomes during and up to 3 months following the end of treatment were also recorded. Tolerability outcomes of interest included grade 3 and 4 adverse effects, and adverse effects requiring Emergency Department (ED) visits, hospital admission or discontinuation of chemotherapy treatment. Grading of adverse events was performed by the treating physician. Adverse events were classified according to the Common Terminology Criteria for Adverse Events (CTCAE) version 5.0. Discontinuation of chemotherapy was defined as administration of fewer treatment cycles than the initially planned total number of adjuvant chemotherapy cycles or the planned cycles in the metastatic setting before a first radiographic evaluation was scheduled.

Means and standard deviations were used as descriptive statistics of continuous parameters and ratios as descriptive statistics of categorical parameters. The Fisher’s exact test or the x^2^ test were used to evaluate differences in categorical parameters of clinical presentation or of pathological characteristics, while the t-test or Analysis of Variance (ANOVA) were used to compare continuous parameters such as mean age. A Receiver Operating Characteristic (ROC) curve analysis with calculation of Area Under the Curve (AUC) and 95% Confidence Intervals (CIs) for the two tools were calculated. The McNemar’s test was used for the comparison of Index4 with the CARG risk categories. Each patient served as its own control with the CARG tool considered the standard and Index4 the experimental comparison. All resulting p-values were considered statistically significant at p < 0.05. The protocol of the research project was approved by the Research Ethics Board of the hospital (Approval # 2019-6-20).

### Ethics approval

The author’s study was performed in line with the principles of the Declaration of Helsinki. Approval was granted by the Ethics Committee of the Sault Area Hospital.

### Consent to participate

This was a study involving no patient intervention and the Sault Area Hospital Ethics Committee has waived individual consent requirement.

## Results

One hundred seventeen patients over age 70 met the inclusion criteria over a 2-year period and participated in the study. The mean age of the cohort was 76.6 years old and 52.1% of the patients were older than 75 years old (Table 1). About two thirds of the patients (63.2%) had localized disease. Most prevalent cancers in the cohort were gastrointestinal cancers (47%), lung cancer (18.8%), breast cancer (15.4%) and gynecologic/genitourinary cancers (10.3%). Most patients (86.3%) had a good ECOG performance status of 0 or 1, while 13.7% of patients had ECOG PS of 2 or 3 (all but 3 patients ECOG PS 2). Radiation therapy during the period of observation in the study was received by 34 patients (29.1%). Concomitant immunotherapy was included with the chemotherapy regimen in 6 patients (5.1%) and concomitant targeted therapies were given in 8 patients (6.8%). About one fourth of patients had lower hemoglobin (< 12 g/dL for men and < 11 g/dL for women) and hypoalbuminemia (Table 2). Low creatinine clearance (< 40 mL/min) and elevated LDH were observed in 9.4% and 15.9% of patients in the cohort, respectively.

**Table 1:** Baseline patient characteristics according to the Index4 score.

Parameter	Category	Total (n = 117)	Index4 = 0 (n = 61)	Index4 ≥ 1 (n = 56)	*p*
Mean age (SD)		76.6 (5)	76.6 (5.3)	76.6 (4.8)	t test, p = 0.98
Age	70–75	56 (47.9)	31 (50.8)	25 (44.6)	0.57
	> 75	61 (52.1)	30 (49.2)	31 (55.4)	
Gender	Male	60 (51.3)	29 (47.5)	31 (55.4)	0.46
	Female	57 (48.7)	32 (52.5)	25 (44.6)	
Stage at time of chemo	1	11 (9.4)	8 (13.1)	3 (5.4)	< 0.0001
	2	25 (21.4)	21 (34.4)	4 (7.1)	
	3	30 (25.6)	25 (41)	5 (8.9)	
	4	43 (36.8)	0	43 (76.8)	
	Localized NOS	8 (6.8)	7 (11.5)	1 (1.8)	
Type of cancer	GI	55 (47)	25 (41)	30 (53.6)	0.02 (χ^2^)
	Breast	18 (15.4)	14 (22.9)	4 (7.1)	
	Lung	22 (18.8)	7 (11.5)	15 (26.8)	
	GU/GYN	12 (10.3)	8 (13.1)	4 (7.1)	
	Other	10 (8.5)	7 (11.5)	3 (5.4)	
Stage at time of chemotherapy	1–3	74 (63.2)	61 (100)	13 (23.2)	
	4	43 (36.8)	0	43 (76.8)	
ECOG PS	0–1	101 (86.3)	61 (100)	40 (71.4)	
	2–3	16 (13.7)	0	16 (28.6)	
Radiation therapy	No	83 (70.9)	38 (62.3)	45 (80.4)	0.04
	Yes	34 (29.1)	23 (37.7)	11 (19.6)	

The last column depicts Fisher’s exact test p values except if otherwise specified.

*BMI* Body Mass Index.

Stage and ECOG PS are among the parameters for the calculations of Index4 and thus by definition no patients had stage 4 disease or ECOG PS of 2 or 3 in the Index4 = 0 group. Percentages are in parentheses except if otherwise specified.

**Table 2:** Laboratory parameters in the groups of Index4 = 0 and ≥ 1.

Parameter	value	Total n = 117	Index4 = 0	Index4 ≥ 1	*p*
Hemoglobin	≥ 12 g/dL (W ≥ 11)	89 (76.1)	50 (82)	39 (69.6)	0.13
	< 12 g/dL (W < 11)	28 (23.9)	11 (18)	17 (30.4)	
Creatinine clearance	≥ 40 mL/min	106 (90.6)	61 (100)	45 (80.4)	
	< 40 mL/min	11 (9.4)	0	11 (19.6)	
Albumin	≥ 35 g/L	87 (74.4)	61 (100)	27 (48.2)	
	< 35 g/L	30 (25.6)	0	29 (51.8)	
LDH	≤ 210 U/L	95 (84.1)	58 (98.3)	37 (68.5)	0.0001
N = 113	> 210 U/L	18 (15.9)	1 (1.7)	17 (31.5)	

The last column depicts Fisher’s exact test p values. Creatinine clearance and albumin are among the parameters for the calculations of Index4 and thus, by definition, no patients had creatinine clearance below 40 mL/min or albumin below35 g/L in the Index4 = 0 group.

Percentages are in parentheses.

**Figure 1:**
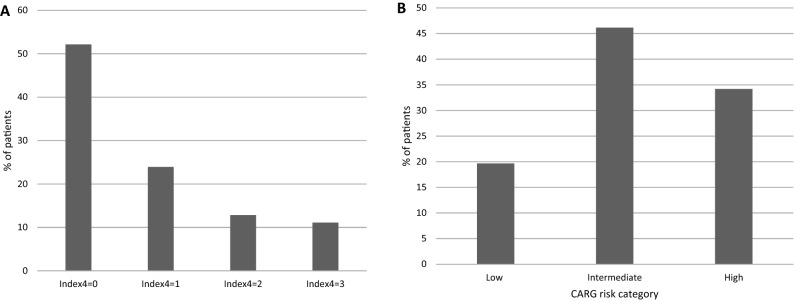
(**A**) Distribution of Index4 scores and (**B**) distribution of CARG risk categories in the cohort of patients included in the study.

Sixty-one patients (52.1%) had an Index4 score of 0, defined as all of the following: ECOG PS 0 or 1, creatinine clearance above 40 mL/min, albumin above 35 g/L and stage 1 to 3 cancer. The remaining 56 patients (47.9%) had an Index4 score above 0. Twenty-eight patients had an Index4 score of 1, 15 patients had a score of 2 and 13 patients had an Index4 score of 3 (Fig. 1). No patients had a score of 4. The two groups of patients with Index4 above 0 and Index4 of 0 had no significant differences in mean age, proportion of patients above age 75 years old or gender (Table 1). Breast, gynecologic and genitourinary cancers were more prevalent in the Index4 score 0 group, while gastrointestinal and lung cancers were more prevalent in the group with Index4 score of 1 or higher. Patients in the Index4 score 0 group received more frequently radiation therapy during the study (37.7% of cases) than patients in the Index4 score of 1 or higher group, who received radiation in 19.6% of cases (Fisher’s exact test p = 0.04, Table 1).

Index4 score above 0 before starting chemotherapy treatment predicted a statistically significant higher rate of ED visits, hospital admissions and discontinuation of chemotherapy treatment. In the group of patients with Index4 score above 0, 61.1% of patients were admitted to ED (with or without subsequent hospitalization) during chemotherapy, 40.7% of patients were admitted to the hospital, and 59.3% of patients discontinued their planned chemotherapy treatment (Table 3). The corresponding percentages for patients with Index4 score of 0 were 33.3% for ED admissions, 19% for hospital admissions and 27% for chemotherapy treatment discontinuation. Patients with Index4 score above 0 who had hospital admissions tended to have a longer hospital stay (mean of 18.5 days versus 12.3 days in admitted patients from the group with Index4 0, p = 0.005). Most common grade 3 or 4 adverse effects observed were neutropenia (29.9%), anemia (13.7%), hypokalemia (10.3%), pain (9.4%) and thrombocytopenia (9.4%) (Supplemental Table 2). The percentage of patients with grade 3 or 4 adverse effects was also higher in patients with Index4 above 0 (64.8% versus 50.8% in patients with Index4 = 0), but this difference did not reach statistical significance.

**Table 3:** Outcomes according to Index4.

Outcome	Total (n = 117)	Index4 = 0 (n = 61)	Index4 ≥ 1 (n = 56)	*p*
Grade 3–4 adverse event				
Yes	67 (57.3)	31 (50.8)	36 (64.8)	0.13
No	50 (42.7)	30 (49.2)	20 (35.2)	
ED visits				
Yes	54 (46.2)	20 (33.3)	34 (60.7)	0.003
No	63 (53.8)	41 (66.7)	22 (39.3)	
Hospital admission				
Yes	34 (29.1)	12 (19.7)	22 (39.3)	0.02
No	83 (70.9)	49 (80.3)	34 (60.7)	
Discontinuation of chemotherapy				
Yes	49 (41.9)	16 (26.2)	33 (58.9)	0.0004
No	68 (58.1)	45 (73.8)	23 (41.1)	

Percentages are in parentheses.

*ED* Emergency Department.

Regarding the distribution of CARG risk scores in the patient cohort, 23 patients (19.7%) had a CARG low risk score, 54 patients (46.1%) belonged to the intermediate CARG risk category and 40 patients (34.2%) were in the high CARG risk category (Table 4, Fig. 1). Mean age of patients was 73.1, 76.2 and 79.2 year-old in the low, intermediate and high CARG categories, respectively (ANOVA p < 0.0001, Table 4). No significant differences were observed between CARG groups in the gender of patients, ECOG PS and percentage of metastatic patients. Breast cancer patients were more commonly included in the low and intermediate risk groups and GI patients in the intermediate and high CARG groups. Lung cancer patients were more commonly in the intermediate group. No differences in the frequency of radiation therapy use in the three groups were present (Table 4). Patients in the high CARG group had more often anemia and lower creatinine clearance compared with the intermediate and low risk groups (Table 5). All four outcomes of interest were significantly more prevalent in the high risk CARG group (Table 6). Grade 3 and 4 adverse effects showed a gradual increase in the three groups, while for ED visits, hospital admissions and chemotherapy discontinuation prevalence in the intermediate group was similar to the low risk group.

**Table 4:** Baseline patient characteristics according to the CARG risk groups.

Parameter	Category	Total (n = 117)	CARG low risk (n = 23)	CARG intermediate risk (n = 54)	CARG high risk (n = 40)	*p*
Mean age (SD)		76.6 (5)	73.1 (4.2)	76.2 (4)	79.2 (5.5)	< 0.0001 (ANOVA)
Age	70–75	56 (47.9)	18 (78.3)	27 (50)	11 (27.5)	0.0004
	> 75	61 (52.1)	5 (21.7)	27 (50)	29 (72.5)	
Gender	Male	60 (51.3)	13 (56.5)	28 (51.9)	19 (47.5)	0.78
	Female	57 (48.7)	10 (43.5)	26 (48.1)	21 (52.5)	
Stage at time of chemotherapy	1	11 (9.4)	2 (8.7)	8 (14.8)	1 (2.5)	
	2	25 (21.4)	6 (26.1)	10 (18.5)	9 (22.5)	
	3	30 (25.6)	5 (21.7)	16 (29.6)	9 (22.5)	
	4	43 (36.8)	7 (30.4)	18 (33.3)	18 (45)	
	Localized NOS	8 (6.8)	3 (13.1)	2 (3.7)	3 (7.5)	
Type of cancer	GI	55 (47)	7 (30.4)	21 (38.9)	27 (67.5)	0.02
	Breast	18 (15.4)	6 (26.1)	9 (16.7)	3 (7.5)	
	Lung	22 (18.8)	7 (30.4)	13 (24.1)	2 (5)	
	GU/GYN	12 (10.3)	0	7 (13)	5 (12.5)	
	Other	10 (8.5)	3 (13.1)	4 (7.4)	3 (7.5)	
Stage at time of chemotherapy	1–3	74 (63.2)	16 (69.6)	36 (66.7)	22 (55)	0.39
	4	43 (36.8)	7 (30.4)	18 (33.3)	18 (45)	
ECOG PS	0–1	101 (86.3)	22 (95.7)	48 (88.9)	31 (77.5)	0.09
	2–3	16 (13.7)	1 (4.3)	6 (11.1)	9 (22.5)	
Radiation therapy	No	83 (70.9)	13 (56.5)	38 (70.4)	32 (80)	0.14
	Yes	34 (29.1)	10 (43.5)	16 (29.6)	8 (20)	

Percentages are in parentheses.

**Table 5:** Laboratory parameters in the groups according to CARG risk category.

Parameter	value	Total (n = 117)	CARG low risk	CARG intermediate risk	CARG high risk	*p*
Hemoglobin	≥ 12 g/dL (W ≥ 11)	89 (76.1)	23 (100)	42 (77.8)	24 (60)	0.004
	< 12 g/dL (W < 11)	28 (23.9)	0	12 (22.2)	16 (40)	
Creatinine clearance	≥ 40 mL/min	106 (90.6)	23 (100)	51 (94.4)	32 (80)	0.04
	< 40 mL/min	11 (9.4)	0	3 (5.6)	8 (20)	
Albumin	≥ 35 g/L	87 (74.4)	21 (91.3)	41 (75.9)	26 (65)	0.06
	< 35 g/L	30 (25.6)	2 (8.7)	13 (24.1)	14 (35)	
LDH	≤ 210 U/L	95 (84.1)	18 (85.7)	44 (84.6)	33 (82.5)	0.9
N = 113	> 210 U/L	18 (15.9)	3 (14.3)	8 (15.4)	7 (17.5)	

Percentages are in parentheses.

**Table 6:** Outcomes according to CARG risk category.

Outcome	Total (n = 117)	CARG low risk	CARG intermediate risk	CARG high risk	*p*
Grade 3–4 adverse event					
Yes	67 (57.3)	9 (39.1)	27 (50)	31 (77.5)	0.004
No	50 (42.7)	14 (60.9)	27 (50)	9 (22.5)	
ED visits					
Yes	54 (46.2)	7 (30.4)	18 (33.3)	29 (72.5)	0.0002
No	63 (53.8)	16 (69.6)	36 (66.7)	11 (27.5)	
Hospital admission					
Yes	34 (29.1)	4 (17.4)	11 (20.4)	19 (47.5)	0.006
No	83 (70.9)	19 (82.6)	43 (79.6)	21 (52.5)	
Discontinuation of chemotherapy					
Yes	49 (41.9)	6 (26.1)	15 (27.8)	28 (70)	< 0.0001
No	68 (58.1)	17 (73.9)	39 (72.2)	12 (30)	

Percentages are in parentheses. *ED* Emergency Department.

A CARG test high category had a sensitivity of 46.3% and a specificity of 82% in predicting grade 3–4 adverse effects (Table 7). A CARG test combined intermediate/high category had an improved sensitivity of 86.6% in predicting grade 3–4 adverse effects while specificity decreased to 28%. Index4 of 1 or above had a sensitivity of 53.7% and a specificity of 60% in predicting grade 3–4 adverse effects (Table 7). In the ROC analysis the AUC of the CARG tool for prediction of grade 3 or 4 adverse effects was 0.83 (95% CI 0.75–0.90) and the AUC of the Index4 tool was 0.76 (95% CI 0.67–0.85). For prediction of ED visits, hospital admissions and discontinuation of chemotherapy, Index4 of 1 or above had slightly better sensitivity but inferior specificity than CARG high category (Table 7). A direct comparison of the two tests showed that an intermediate or high CARG was superior in predicting grade 3 or 4 toxicity than Index4 of 1 or above (Table 8). For prediction of the 3 other outcomes the two tools performed similar, while Index4 of 0 was superior in predicting absence of outcomes (Table 8).

**Table 7:** Sensitivities, specificities, positive predictive values (PPV), negative predictive values (NPV) and accuracy for prediction of adverse chemotherapy outcomes of the CARG and Index4 tools.

Outcome	Test result	Sensitivity (%)	Specificity (%)	PPV (%)	NPV (%)	Accuracy (%)
Grade 3–4 toxicity	CARG high	46.3	82	77.5	53.2	61.5
	CARG intermediate/high	86.6	28	61.7	60.9	61.5
	Index4 ≥ 1	53.7	60	64.3	49.2	56.4
ED visits	CARG high	53.7	82.5	72.5	67.5	69.2
	Index4 ≥ 1	63	65.1	60.7	67.2	64.1
Hospital admission	CARG high	55.9	74.7	47.5	80.5	69.2
	Index4 ≥ 1	64.7	59	39.3	80.3	60.7
Discontinuation of chemotherapy	CARG high	57.1	82.3	70	72.7	71.8
	Index4 ≥ 1	67.3	66.2	58.9	73.8	66.7

*ED* Emergency Department.

**Table 8:** Comparison of CARG with Index4.

Outcome	Comparison	McNemar’s test p	Odds ratio (95% CI)
Grade 3–4 toxicity	CARG high versus Index4 ≥ 1	0.44	1.45 (0.63–3.46)
	CARG intermediate/high versus Index4 ≥ 1	0.0001	0.12 (0.02–0.39)
Absence of grade 3–4 toxicity	CARG low versus Index4 = 0	0.002	5 (1.67–20.11)
	CARG low/intermediate versus Index4 = 0	0.01	0.15 (0.01–0.68)
ED visits	CARG high versus Index4 ≥ 1	0.38	1.62 (0.62–4.52)
No ED visits	CARG low versus Index4 = 0	0.0001	9.33 (2.88–47.9)
Hospital admission	CARG high versus Index4 ≥ 1	0.62	1.42 (0.49–4.42)
Absence of hospital admission	CARG low versus Index4 = 0	0.0001	8.5 (3.03–32.9)
Chemotherapy discontinuation	CARG high versus Index4 ≥ 1	0.38	1.62 (0.62–4.5)
Chemotherapy completion	CARG low versus Index4 = 0	0.0001	8 (2.83–31.1)

Odds ratios (ORs) below 1 favor CARG and ORs above 1 favor Index4.

*CI* confidence interval. *ED* Emergency Department.

## Discussion

Chemotherapy remains an integral part of cancer treatment, extending survival in both localized and metastatic cancers. Adverse effects of these treatments can be severe, especially for older cancer patients with comorbidities and decreased performance status or even for geriatric patients without comorbidities but with decreased organ reserves due to the normal aging process^8^. Thus, an important conundrum in clinical oncology is to identify older cancer patients who could tolerate a chemotherapy treatment and to differentiate them from patients with similar age who would not be expected to tolerate such therapy without serious adverse effects. Such ability to predict chemotherapy tolerance would allow geriatric patients to receive and benefit from chemotherapy while withholding or modifying the intensity of chemotherapy in the case of patients predicted to be intolerant.

A few tools have been proposed for prediction of chemotherapy adverse effects in the geriatric oncology population. The most established predictor has been introduced by the Cancer and Aging Research Group (CARG) and has been endorsed by ASCO^3^. This tool was able to accurately predict grade 3 and 4 toxicities in patients with lung, breast, gynecologic or genitourinary cancers over the age of 65 who received chemotherapies^5^. Patients were classified in three grade 3–4 toxicity risk categories based on a score ranging from 0 to 19. Patients at the low risk category (score 0 to 5) had a grade 3–4 adverse effects prevalence of 30%, while patients in the intermediate risk (score 6 to 9) and the high risk (score 10 to 19) groups had a grade 3–4 toxicity prevalence of 52% and 83%, respectively. However, the CARG predictor is less suitable for busy oncology practices with scarce resources as it requires a calculation based on 11 parameters with different weights factored in.

We previously devised an alternative simpler predictive tool for use in geriatric cancer patients for prediction of chemotherapy toxicities and hospital admissions^7^. This predictor, Index4, is easily calculated by 2 laboratory values, albumin and creatinine clearance, as well as ECOG PS and stage of the cancer. In the current investigation, we analyzed a cohort of cancer patients over the age of 70 years old scheduled to receive chemotherapy treatment, with the aim to predict adverse outcomes of treatment, including grade 3 and 4 adverse effects, ED visits, hospital admissions and premature treatment discontinuation. Overall, the ability to predict grade3 and 4 toxicities in this cohort was moderate with an accuracy of 56% to 61%. An association of grade 3 and 4 toxicities was shown with the high risk categories of the CARG tool (p = 0.004), while Index4 of 1 or above, although associated with a higher rate of grade 3 and 4 toxicities (64.8% versus 50.8% grade 3 and 4 toxicities rate in patients with Index4 of 0) was not statistically significant (p = 0.13). Prediction of ED visits, hospital admissions and treatment discontinuation was also moderately accurate and the two tools showed comparable performance. In addition, an Index4 of 0 performed better than the low CARG category for predicting the absence of ED visits, hospital admissions and chemotherapy discontinuation. Given the ease of use and comparable if not better performance for exclusion of adverse outcomes, Index4 could be an alternative to CARG tool. Preferably, further validation would increase the confidence for this conclusion. Consistent with the current study, the CARG tool had been previously found to perform similarly to a tool called Vulnerable Elders Survey 13 (VES-13) as well as with oncologist’s judgement for prediction of grade 3 and higher toxicity of chemotherapy in men over the age of 65 receiving docetaxel for prostate cancer^9^.

It is apparent that there is room for improvement in the predictive tools of chemotherapy adverse effects in the geriatric cancer population. The CARG tool that is currently one of the recommended tools, is more accurate than Index4 (accuracy 61.5% versus 56.4% accuracy of Index4) for the primary prediction of grade 3 and 4 adverse effects, for which outcome it was created, but is not performing better than Index4 for other adverse outcomes of interest. Besides, the aforementioned impracticality, the CARG predictor presents other disadvantages that include the fact that some of the functional parameters used are subjective and rely on patient perceptions. Two other factors included in the CARG score calculation relate to the number and dose of chemotherapy drugs used, which would be among the parameters to modify according to the prediction calculation. The Index4 tool was created with the aim of being practical and specifically seeks to predict adverse effects without the need for functional parameters. Thus, it could potentially be improved, without becoming impractical, by the incorporation of a functional parameter, in addition to ECOG PS. Moreover, Index4 includes only four objectively measurable clinical and laboratory parameters and is not based on parameters related to the chemotherapy treatment to be used. Thus, it avoids the confounding that may be produced by the chemotherapy intensity parameters included in the CARG tool calculations.

This research compares the Index4 predictor with the CARG tool and has not investigated other tools such as the CRASH tool or other less commonly used tools. The CRASH predictor, which is an alternative tool to CARG tool suggested by the ASCO guidelines for prediction of toxicity in geriatric cancer patients, is also a rather complicated tool to calculate, with no clear advantage over the CARG tool. Another limitation of the current study includes the fact that it describes a single center experience and it is unknown if the two tools will perform similarly in other practice settings. Moreover, radiation therapy, immunotherapy and targeted therapies have been given concomitantly with chemotherapy in 29.1%, 5.1% and 6.8% of the patients in the study and their associated toxicities may have confounded the observed toxicities in a sub-set of patients.

Index4 cannot claim superiority of prediction of grade 3 and 4 toxicities from chemotherapy in geriatric patients and in fact performs worse in that regard, although not significantly so (For example, the AUC 95% CIs for prediction of grade 3/4 toxicities of the two tools overlap). For other outcomes (ED and hospital admissions and chemotherapy discontinuation) the predictive power of the two tools is super-imposable. On the other hand a low Index4 score predicts better than the CARG low risk category the lack of grade 3/4 toxicities, which could be of significant clinical value. Moreover, the simplicity of Index4 may help increase its uptake in the oncology clinic with resulting improvement in chemotherapy outcomes in the geriatric population. Improved prediction of chemotherapy toxicity and other adverse outcomes in elderly cancer patients could significantly improve cancer and treatment outcomes in this population and improve quality of life as well health system resources allocation. A prerequisite for improved prediction is to have a tool that is practical enough to be integrated to everyday clinical practice without excessively increasing burden. The Index4 tool fulfils the practicality prerequisite and could constitute a starting point for attaining the unmet need of chemotherapy adverse effects prediction in geriatric oncology.

## Supplementary Information

Supplementary Tables.

## Data Availability

All data generated or analysed during this study are included in this published article [and its Supplementary Information files].
