# Supplementary material for: An evaluation of the Index4 tool for chemotherapy toxicity prediction in cancer patients older than 70 years old
Source: Sci Rep. 2023 Jan 19;13:1082. doi: 10.1038/s41598-023-28309-5 (PMC9852555; doi:10.1038/s41598-023-28309-5)
Supplement: Supplementary file 1 — Supplementary Tables. [file 41598_2023_28309_MOESM1_ESM.docx]

Supplemental Table 1. Parameters and cut-offs used to calculate the CARG and Index4 predictors. Possible range for the CARG tool is 0 to 23 and for Index4 is 0 to 4.

| CARG |  | Index4 |  |
| --- | --- | --- | --- |
| Parameter and cut-off | Points attributed | Parameter and cut-off | Points attributed |
| Age ≥ 72 years old | 2 | ECOG PS > 1 | 1 |
| GI or GU cancer | 2 | Albumin < 35 g/ L | 1 |
| Standard dose chemotherapy | 2 | Creatinine Clearance< 40ml/ min | 1 |
| Polychemotherapy (≥ 2 drugs) | 2 | Stage 4 | 1 |
| Hemoglobin <11 g/ dL (W<10) | 3 |  |  |
| Creatinine Clearance< 34ml/ min | 3 |  |  |
| Hearing fair or worse | 2 |  |  |
| ≥ 1 falls in the last 6 months | 3 |  |  |
| Unable to take medications or need help | 1 |  |  |
| Limited in walking 1 block | 2 |  |  |
| Decreased social activity | 1 |  |  |

Supplemental Table 2. Most common grade 3-4 adverse effects in the study cohort.

| System | Adverse effect | Number of patients | % of patients with grade 3-4 adverse effects | % of all patients |
| --- | --- | --- | --- | --- |
| Hematologic | Leukopenia/ Neutropenia | 35 | 52.2 | 29.9 |
|  | Anemia | 16 | 23.9 | 13.7 |
|  | Thrombocytopenia | 11 | 16.4 | 9.4 |
| Infectious | Febrile neutropenia/ Sepsis | 3 | 4.5 | 2.6 |
| Respiratory | Dyspnea | 3 | 4.5 | 2.6 |
| Metabolic | Hypokalemia | 12 | 17.9 | 10.3 |
| Gastrointestinal | Mucositis/ enteritis | 3 | 4.5 | 2.6 |
|  | Diarrhea | 4 | 6 | 3.4 |
| Constitutive | Fatigue | 5 | 7.5 | 4.3 |
|  | Pain | 11 | 16.4 | 9.4 |
